# Coating of upconversion nanoparticles with silica nanoshells of 5–250 nm thickness

**DOI:** 10.3762/bjnano.10.231

**Published:** 2019-12-09

**Authors:** Cynthia Kembuan, Maysoon Saleh, Bastian Rühle, Ute Resch-Genger, Christina Graf

**Affiliations:** 1 Institut für Chemie und Biochemie, Physikalische und Theoretische Chemie, Freie Universität Berlin, Takustraße 3, D-14195 Berlin, Germany; 2 Bundesanstalt für Materialforschung und -prüfung (BAM), Richard-Willstätter-Str. 11, D-12489 Berlin, Germany; 3 Hochschule Darmstadt - University of Applied Sciences, Fachbereich Chemie- und Biotechnologie, Stephanstr. 7, D-64295 Darmstadt, Germany

**Keywords:** reverse microemulsion, silica coating, stepwise growth, thick shells, upconversion nanoparticles

## Abstract

A concept for the growth of silica shells with a thickness of 5–250 nm onto oleate-coated NaYF_4_:Yb^3+^/Er^3+^ upconversion nanoparticles (UCNP) is presented. The concept enables the precise adjustment of shell thicknesses for the preparation of thick-shelled nanoparticles for applications in plasmonics and sensing. First, an initial 5–11 nm thick shell is grown onto the UCNPs in a reverse microemulsion. This is followed by a stepwise growth of these particles without a purification step, where in each step equal volumes of tetraethyl orthosilicate and ammonia water are added, while the volumes of cyclohexane and the surfactant Igepal^®^ CO-520 are increased so that the ammonia water and surfactant concentrations remain constant. Hence, the number of micelles stays constant, and their size is increased to accommodate the growing core–shell particles. Consequently, the formation of core-free silica particles is suppressed. When the negative zeta potential of the particles, which continuously decreased during the stepwise growth, falls below −40 mV, the particles can be dispersed in an ammoniacal ethanol solution and grown further by the continuous addition of tetraethyl orthosilicate to a diameter larger than 500 nm. Due to the high colloidal stability, a coalescence of the particles can be suppressed, and single-core particles are obtained. This strategy can be easily transferred to other nanomaterials for the design of plasmonic nanoconstructs and sensor systems.

## Introduction

Lanthanide-based nanocrystals have gained importance as inorganic optical reporters in recent years [1–3]. The doping of inorganic host NaYF_4_ matrices with different optically active lanthanide ions can result in so-called upconversion nanoparticles (UCNP) which can absorb photons of lower energy (e.g., near-infrared (NIR) light) and emit photons of higher energy (e.g., visible light) via a two- or multiphoton upconversion mechanism involving several energy transfer steps [2–5]. Advantages of UCNPs compared to organic dyes or other inorganic nanoscale reporters are the emission of a multitude of characteristic narrow emission bands in the ultraviolet/visible/NIR upon excitation in the NIR range where light absorption and scattering from biological tissues is minimal as well as long fluorescence lifetimes in the microsecond range that are insensitive to oxygen, a high chemical stability and a low cytotoxicity [6–7]. This makes UCNPs attractive for applications in the life sciences [8–11]. Some of the most frequently used UCNPs are NaYF_4_-based nanoparticles (NPs) with Yb^3+^ as the light absorbing sensitizer and Er^3+^ as the emitting activator [12–15]. Monodisperse UCNPs with relatively high quantum yields are typically prepared in organic solvents at high temperatures using hydrophobic capping agents such as oleic acid [16–17]. Life sciences applications of these NPs require to render them water-dispersible using either ligand exchange or encapsulation procedures [2,13,18–19]. This can be similarly necessary for applications in plasmonics or chemical sensing [20–21].

One of the most versatile ways to protect the surface of NP, making hydrophobic particle surfaces hydrophilic and simultaneously providing functional groups for subsequent covalent attachment of, e.g., biomolecules, is the coating of their surface with silica shells [22–23]. Additionally, optically transparent silica shells have many other advantages such as chemical inertness, high thermal stability, low cytotoxicity, high biocompatibility and tunable porosity [22–24]. An important parameter for all shelling procedures is the precise control of the shell thickness while preventing or at least minimizing the formation of additional seeds from the shelling material. Numerous approaches have been investigated for the growth of silica shells on inorganic NPs like the Stöber synthesis and the reverse microemulsion method. The Stöber method refers to the process of preparing silica via the hydrolysis and condensation of tetraethyl orthosilicate (TEOS) within an alcohol–ammonia–water system [25]. Related methods are widely used for coating NPs that are dispersible in polar media [26–27]. Modified Stöber processes, in which TEOS is continuously added to seeds in a growth solution, allow for the growth of large, monodisperse NPs in a single step, provided the seed NPs are well dispersed in the growth solution [28–29]. A versatile approach for growing silica shells onto inorganic NPs that cannot be dispersed in polar media is the reverse microemulsion technique [22–23,30–43]. In a reverse microemulsion, the aqueous solution is confined in uniform, nanosized droplets that are stabilized by a surfactant such as a polyoxyethylene (5) nonylphenylether (trade name Igepal^®^ CO-520) and distributed in the continuous nonpolar phase [44]. The ratio between the aqueous components and the surfactant determines the size of these droplets [30], which act as nanoreactors. For the polycondensation of precursors such as TEOS, ammonia usually acts as a catalyst [43]. This technique allows for the formation of uniform silica shells on individual particle cores [23,40–41].

Up to now, syntheses of UCNPs with relatively thin silica shells (mostly 1–10 nm) have mostly been reported. Li et al. presented the first approach to coat oleate-stabilized UCNPs via the reverse microemulsion technique in 2008 [42–43]. However, for certain applications such as sensing and plasmonics, a thicker silica shell is desired that can be loaded with sensor molecules or used as spacer for the plasmonic enhancement of the emission of UCNPs by gold or silver shells [45]. Moreover, since UCNPs can release rare earth metal and fluoride ions to some extent into the surrounding medium [46], which can cause toxic effects, a thick silica shell could act as protective coating [46].

For silica shells grown onto iron oxide NPs using an inverse microemulsion, it was shown that the thickness of the shell increases as the amount of TEOS increases, while core-free silica NPs appear when the TEOS content exceeds the threshold of homogeneous nucleation [36,39,47–49]. Typically, a maximum diameter <50 nm can be reached with this technique [23]. Microemulsion growth processes are usually slow and laborious as one has to control the water-to-surfactant ratio to prevent the formation of core-free silica NPs. In this respect, combining this technique with Stöber growth can be advantageous [50]. For example, Katagiri managed to further grow silica-coated Fe_3_O_4_ particles with a thin shell to a diameter >100 nm by a similar procedure [23].

In this work, we present an approach for growing a silica shell with an adjustable thickness between 5 and 250 nm onto oleate-coated NaYF_4_:Yb^3+^/Er^3+^ UCNP. This coating procedure comprises the growth of a silica shell via a reverse microemulsion method to shell thicknesses of about 40–50 nm, followed by the growth of a thick silica layer by continuously adding TEOS in a Stöber-like growth step. Thereby, particle aggregation, which can occur during a Stöber-like growth process, and the formation of NPs from the shell material can be elegantly prevented, and monodisperse particles with just one UCNP core in the center coated by a thick silica shell are obtained. This method should also be suitable for other NPs with hydrophobic surfaces dispersed in an apolar solvent independent of their chemical composition.

## Results and Discussion

**Figure 1:**
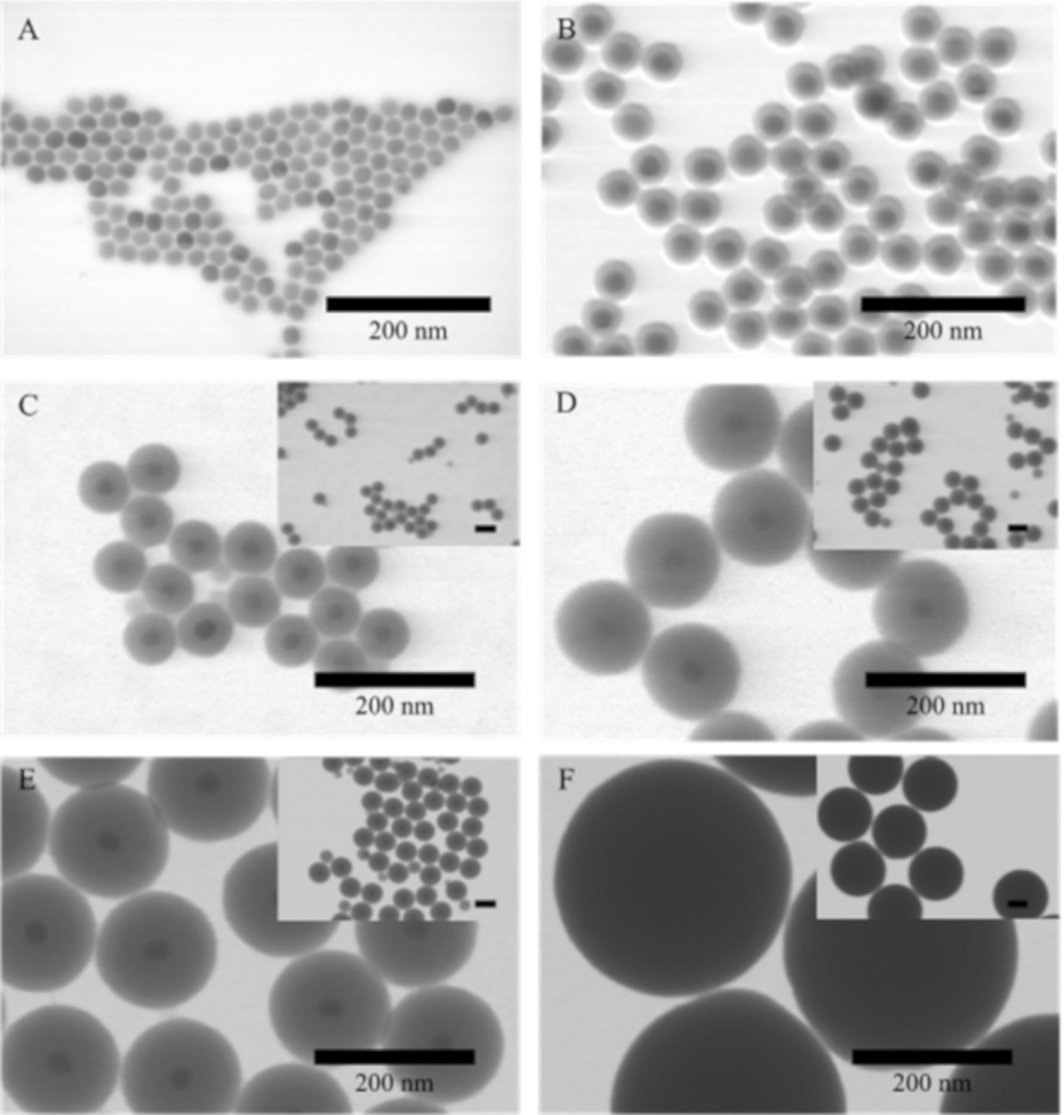
TEM images of (A) NaYF_4_:(Yb,Er) cores (C1; diameter: 24 ± 1 nm) and (B) the same core after coating with the first thin silica shell (C1_1S, shell thickness: 7 ± 1 nm). Image (C) shows the same UCNP cores after the second silica coating step (C1_2S, shell thickness: 18 ± 2 nm), (D) after the third silica coating (C1_3S, shell thickness: 35 ± 2 nm), (E) after the fourth shell silica coating (C1_4S, shell thickness: 44 ± 2 nm) and (F) after the fifth silica growth step (C1_5S, shell thickness: 149 ± 8 nm). The first to the fourth silica shell were grown with the reverse microemulsion method, whereas the fifth shell was grown using a modified Stöber growth. The scale bar in the insets of panels (C–F) represents 100 nm.

The core particles used in this study, i.e., oleate-capped UCNPs with a NaYF_4_ host structure and doped with 18% Yb and 2% Er, were synthesized by a thermal decomposition method [16] yielding spherical particles of low polydispersity. A typical scanning transmission electron microscopy (STEM) image is shown in Figure 1A. The diameter of the UCNP@SiO_2_ core–shell particles was obtained from these STEM images, and the corresponding hydrodynamic diameters were measured by dynamic light scattering (DLS, see below in Table 1). Although large, core-free silica particles can easily be obtained by Stöber-like growth processes [28], and the controlled growth of silica particles to large monodisperse particles with a precisely predetermined diameter is well-established, a direct Stöber growth of silica shells on hydrophobic particles in a nonpolar solvent is not feasible. Aiming at the development of a synthesis providing maximum growth of a silica layer in a single step without producing UCNP-free silica particles as side products, a thin silica layer was grown first onto the particles via a reverse microemulsion process in cyclohexane with Igepal CO-520 as surfactant and ammonia as a catalyst. In such a reverse microemulsion, the size and the number of the aqueous domains, i.e., the water pools inside the micelles, are determined by the ratio of ammonia water to Igepal CO-520, often denoted as the R-value [30,36,51–52]. Several authors suggested that for an optimal growth process where particles with multiple cores as well as coreless particles are absent, the number of micelles has to ideally match the number of particles [36,47]. If in the course of this process the silica shell becomes thicker, ammonia water and surfactant must be added accordingly in order to balance the particle growth, while suppressing the formation of new micelles [23]. Ding et al. linked these considerations to the theory of LaMer [36]. According to the LaMer theory, heterogeneous nucleation occurs when the supersaturation of the growth species is below the homogeneous nucleation threshold but above the heterogeneous nucleation threshold, while a higher supersaturation (above the homogeneous nucleation threshold) leads to simultaneous heterogeneous and homogeneous nucleation. In general, the processes leading to homogeneous and heterogeneous nucleation and growth in such reverse microemulsion systems are complex and depend on numerous factors. Our considerations for the growth of thick silica shells on UCNPs are based on the models presented by Ding et al. [36] and Katagiri et al. [23] for silica-coated iron oxide NP.

For UCNPs with a diameter of 24 ± 2 nm and a particle concentration of 3 g/L, with an ammonia water-to-surfactant weight ratio of 1:9.5 or a molar ratio of 1:2.7 and an ammonia water concentration of 1.7 ± 0.5 wt % in cyclohexane almost no core-free particles (<1%) are formed. For the desired control of the growth process and hence shell thickness, a relatively low ammonia water-to-surfactant ratio was used as this slows down the hydrolysis of TEOS and thus shell growth. A general scheme of the growth of the initial silica layer on the UCNPs with this reverse microemulsion process is shown in Figure S1 in Supporting Information File 1. According to the mechanism of silica growth reported for oleate-functionalized iron oxide NP, the oleate ligands on the NP surface are at least partly exchanged for the surfactant as well as the hydrolyzed TEOS upon addition of the oleate-functionalized NPs to the Igepal CO-520–cyclohexane system [36,47]. A similar process is assumed for the oleate-capped UCNPs. As the size of our UCNPs was 2–3 times larger than the size of the iron oxide NPs [23,36], and their number concentration was 8 and 1.6 times higher than the ones used in [36] and [23], respectively, we used a higher concentration of Igepal CO-520. In our case, even with a significantly lower ratio of surfactant to particle surface (1.6 mol/m^2^ in the present case, 9.5 mol/m^2^ and 5.2 mol/m^2^ in the cases of Ding et al. [36] and Katagiri et al. [23], respectively) no silica particles with multiple UCNP cores were formed. Based on these considerations, we also used a lower value of the ammonia water-to-surfactant (*R*) weight ratio for the first silica shell growth steps (1:9.4 in the present case; compared to 1:2.7 in the work of Ding et al. [36] and 1:6.1 in the work of Katagiri et al. [23]).

For further shell growth, even a slightly lower Igepal CO-520 concentration in cyclohexane (from 16 wt % for the first to 14 wt % for the subsequent shell growth steps) and an increased ammonia water concentration (from 1.7 wt % for the first and 3.3 wt % for the subsequent shell growth steps) were employed, raising the R-value from 1:9.5 to 1:4.3. In this way, the number of micelles was kept constant, and their diameters were adjusted so that they are large enough to host the growing core–shell particles.

After an initial silica shell of 5–10 nm was coated onto the UCNP, a further growth by a Stöber-like growth process was attempted, i.e., the particles were redispersed in ethanol, and ammonia water, water and TEOS were added. However, these attempts resulted in samples where most of the particles are grown together as well as in the formation of core-free silica particles. Figure S2 in Supporting Information File 1 shows UCNPs with a diameter of 20 ± 2 nm (sample C2_1S) initially coated with an 11 ± 1 nm silica shell followed by the Stöber-like regrowth. Similar findings were also obtained for larger UCNP. It turned out in several preliminary experiments conducted by the same procedure that the zeta potential of the particles after the initial silica growth in the reverse microemulsion was always only around −20 mV, which explains the low colloidal stability of these particles. The latter was also confirmed by the rather high hydrodynamic diameter of the particles derived from DLS compared to the diameter obtained by STEM (see below in Table 1). A similarly low colloidal stability of the NPs coated with silica in reverse microemulsions was reported before [53–56] and attributed to the presence of the Igepal CO-520 on the NP surface. However, extensive purification of the particles after the growth of the first silica shell by repeated centrifugation and redispersion in ethanol did not significantly alter their zeta potential.

**Figure 2:**
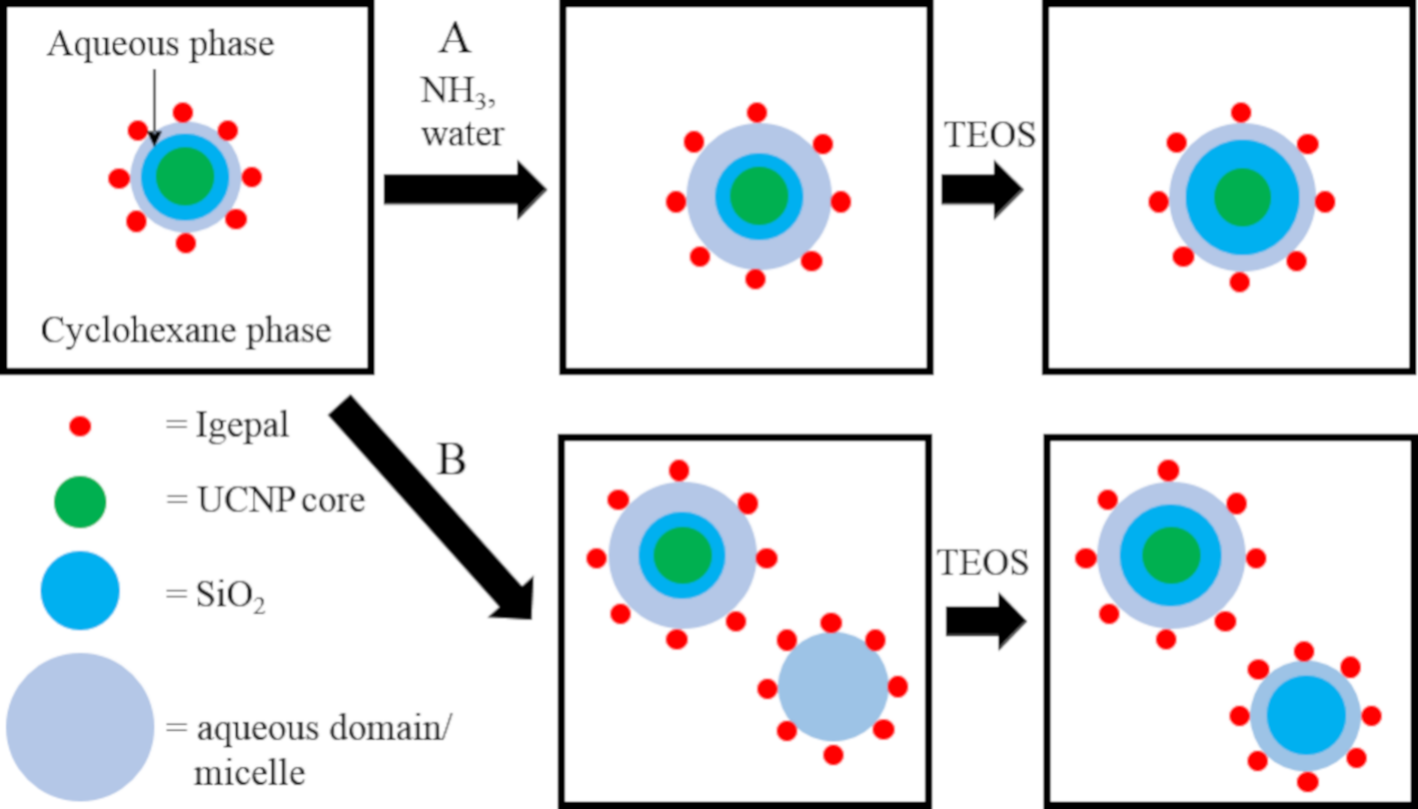
Scheme of the reverse microemulsion synthesis for growing thicker silica shells after a first silica coating on the UCNP. Path A describes the controlled growth of the silica shell, while path B depicts the formation of core-free silica particles due to TEOS hydrolysis in core-free micelles caused by an increasing water-to-surfactant (*R*) ratio due to ammonia water addition during the further steps of silica shell growth.

For this reason, further silica shell growth was performed in a reverse microemulsion. For this procedure, initially, the concept for growing larger silica shells on oleate-coated iron oxide NPs introduced by Ding et al. was adapted for the UCNPs [36] and used for a step-wise growth process (Figure 2). According to this model, the controlled addition of ammonia water along with increasing the amount of surfactant corresponding to the size of the (silica-coated) core should lead to slow hydrolysis of TEOS and consequently a well-controlled growth of the silica shell yielding a thin silica shell (Figure 2, path A) [30]. In contrast, if the ammonia water concentration is quickly raised from a low R-value, the volume of the water domain in the micelles increases. This causes an increase in the hydrolysis rate and the formation of new empty micelles and promotes the formation of new silica particles and uncontrolled silica growth (see Figure 2 path B) [36]. In the STEM image in Figure S3 in Supporting Information File 1, a sample is shown where the R-value was only 1:2.2, and consequently, many core-free silica particles were formed. Hence, for the further shell growth, especially for a silica shell thickness exceeding 10 nm, the R-value was adjusted to 1:4.3 to keep the aqueous domain large enough for the growth of thicker silica shells but small enough to suppress the formation of core-free silica particles. In the following, the amount of each chemical for the further steps of the silica shell growth is discussed. For a second silica shell with the thickness *t*_2_, the volume of TEOS (*V*_T_) was calculated for a given mass *m*_UCNP_ of uncoated UCNP cores with diameter *d*_U_ according to Equation 1 assuming 100% conversion of TEOS to SiO_2_ and the absence of any secondary nucleation:

[1] $$V_{\text{T}}=\left(\frac{{\left(\frac{d_{\text{U}}}{2}+t_{1}+t_{2}\right)}^{3}-{\left(\frac{d_{\text{U}}}{2}+t_{1}\right)}^{3}}{{\left(\frac{d_{\text{U}}}{2}\right)}^{3}}\right)\frac{\rho_{\text{S}}\cdot m_{\text{U}}\cdot M_{\text{T}}}{\rho_{\text{U}}\cdot M_{\text{S}}\cdot \rho_{\text{T}}},$$

where *t*_1_ is the thickness of the first silica shell, ρ_S_ is the density of colloidal silica (2 g/cm^3^), ρ_U_ is the density of the UCNP cores (4.21 g/cm^3^), *M*_T_ is the molar mass of TEOS (208.32 g/mol), *M*_S_ is the molar mass of SiO_2_ (60.08 g/mol) and ρ_T_ is the density of TEOS (0.94 g/cm^3^). The added volume of ammonia water always matched that of the added TEOS. The volume of cyclohexane was calculated for each growth step such that the ammonia water concentration in cyclohexane was 3.3 ± 0.1 wt %. The concentration of Igepal CO-520 was kept constant at 14 ± 1 wt % in cyclohexane throughout all growth steps in the reverse microemulsion, resulting in an R-value for the further shell growth of 1:4.3 (weight ratio) or 1:6.0 (molar ratio). The R-value was increased compared to the growth of the first shell to keep the water domain large enough for the increasing size of the particles while maintaining a constant ratio of the number of micelles and the number of particles. The control of the Igepal CO-520 concentration prevents the formation of core-free micelles and provides the particles with sufficiently large water domains for further TEOS hydrolysis. If the surfactant concentration is too high while the concentrations of the other reactants is constant or too low, new micelles are formed, which can facilitate the formation of core-free silica particles. Ding et al. used a slightly lower concentration of ammonia water (1 wt % compared to 1.7 ± 0.5 wt % in the present work) and a lower surfactant concentration (5.6 wt % compared to 16 wt %) for the growth of a single shell, corresponding to an R-value of 1:5.5 for iron oxide core particles with diameters of 12.2 nm. Under these conditions, they were able to vary the added amount of TEOS in a range of 75–600 μL so that they could adjust the thickness of the silica shell. Katagiri et al. used ammonia water and surfactant concentrations of 0.83 wt % and 5.1 wt %, respectively, for iron oxide particles with diameters of 10 nm (*R* = 1: 6.1 in weight ratio). They used the same concentration of both components also for the stepwise growth of a thicker silica shell. This concentration was significantly lower than the concentration (16 wt %) used in this work, especially in the case of Igepal CO-520. This difference could explain why the maximum size of the core–shell particles did not exceed 50 nm before core-free particles started to form in the experiments conducted by Katagiri and co-workers [23]. These studies and their comparison underline the many possibilities of varying the parameters of the shell growth in the reverse microemulsion approach. However, we could show that the reported R-value can be utilized to synthesize a wide range of silica shells with different thicknesses.

In a typical example, a UCNP core (NaYF_4_ doped with Yb and Er; core sample C1) with a diameter of 24 ± 1 nm was coated with silica shells through a stepwise reverse microemulsion synthesis. The silica shell thickness increased here in four growth steps from 7 to 44 nm (Figure 1). The terminology used for each shell is C1_1S for the first shell, C1_2S for the second shell and so on.

For all growth steps, the measured shell thicknesses from STEM agree relatively well with the calculated shell thicknesses (Table 1 and Table S1, Supporting Information File 1). This supports that all TEOS grows as SiO_2_ on the existing core particles. The observation that the measured shell thickness was slightly larger than the calculated one can be explained by the fact that the total mass of the particles, including the oleate ligands, was used for the calculations. The oleate ligands are, however, exchanged during shell growth in the inverse microemulsion [36,47]. The oleate content for particles of this size was in the range of 5–10 wt % as shown by thermogravimetric analysis [57]. The z-average values of the samples after the first and second shell indicate low colloidal stability of the particles, which is also supported by the high PDI values suggesting partial aggregation (Table 1). Repeated centrifugation and redispersion in ethanol were carried out in an attempt to improve the colloidal stability by removing the remaining surfactant from the surface. However, this procedure did not increase the stability of the particles. This colloidal instability of NPs with thin silica shells obtained from the reverse microemulsion syntheses was also reported by several other authors before [53–56]. In contrast to these findings, after the third and fourth steps of shell growth, the particles have a relatively low PDI, and the z-average diameters match the radii obtained from STEM much more closely, indicating their high colloidal stability. The zeta potential becomes increasingly more negative with the growth of thicker silica shells. The particles after the second step of the silica growth (C1_2S) have a zeta potential of −32 ± 1 mV (Table 1), which decreases to −41 ± 1 mV after the formation of the third shell. The samples after the fourth silica shell growth step have a zeta potential of −45 ± 1 mV, which is in the range typically found for particles from Stöber-like growth processes [58]. This increasingly more negative zeta potential likely arises from a decrease of the surface concentration of Igepal CO-520 on the growing silica-coated particles and was repeatedly found in this work. Due to the increased colloidal stability, it was then possible to continue to further grow the shells in a Stöber-like growth process. Under these conditions, the silica growth itself is much faster than in a reverse microemulsion [59]. Moreover, modified Stöber processes where TEOS is continuously added allow for the growth of silica layers that are several hundred nanometers thick at high precision in one step [28]. The particles were transferred into ethanol with a rather high ammonia water concentration (14.4 wt %). A fifth shell was then grown on sample C1_4S by continuously adding TEOS. In this way, the particles could be grown directly from 112 ± 4 nm diameter to a size of 321 ± 16 nm (sample C1_5S). A z-average value that is similar to the diameter measured in STEM and a relatively low PDI of this sample (Table 1) indicate the formation of monodisperse particles (Figure 1F). This result shows that the Stöber method allowed for a significant increase in the particle size within one step. In the case of sample C1_4S, the particle volume could be grown more than 23-fold.

**Table 1:** Overview of the size, silica shell thickness, z-average, PDI and zeta potential of each silica-coated sample. DLS of the core was performed in cyclohexane, while the silica-coated samples were measured in ethanol, and the zeta potential was measured in water.

sample	shell	total diameter (STEM)	silica shell thickness (STEM)	z-average	PDI	zeta potential
		[nm]	[nm]	[nm]		[mV]

C1	core	24 ± 2	0	44 ± 2	0.360 ± 0.020	n.d.
C1_1S	1st	38 ± 2	7 ± 2	89 ± 2	0.090 ± 0.020	n.d.
C1_2S	2nd	59 ± 3	18 ±4	98 ± 2	0.110 ± 0.030	−32 ± 1
C1_3S	3rd	93 ± 4	35 ± 4	116 ± 2	0.013 ± 0.005	−41 ± 1
C1_4S	4th	112 ± 4	44 ± 4	137 ± 2	0.040 ± 0.010	−45 ± 1
C1_5S (Stöber)	5th	321 ± 16	149 ± 16	376 ± 9	0.095 ± 0.020	−37 ± 1

Core-free silica particles were formed in one growth step during the stepwise growth process (Figure 1C). To obtain further shells of the same thickness as the initial silica shell, several smaller growth steps were carried out in the later syntheses described in the following. Smaller amounts of TEOS were added per step, and the other chemicals were also added in correspondingly smaller steps (Figure S4 in Supporting Information File 1). The ammonia water and Igepal CO-520 concentrations as well as the R-values were the same as used for the initial syntheses. Smaller growth steps helped to prevent possible minor new nucleation of silica due to a locally too high TEOS concentration. Moreover, smaller growth steps have the advantage that accidentally formed secondary nuclei can be removed more easily by centrifugation since the difference between newly formed particles and the core–shell particles is larger. When the zeta potential was sufficiently negative (−50 ± 8 mV), the microemulsion was broken, and the particles were transferred to an ethanol solution containing ammonia water. Subsequently, a modified Stöber growth was performed where TEOS was continuously added over several hours with a peristaltic pump. In this way, particles with a diameter exceeding 500 nm and a narrow size distribution could be grown within one step (Figure S4H−J in Supporting Information File 1).

Overall, the growth of a silica shell with the reverse microemulsion method initially decreased the colloidal stability of the particles, as shown by the diameter of the UCNPs as determined by TEM and the deviating z-average and low zeta potential values. The particle stability could be increased by growing a thicker silica shell through a further stepwise use of the reverse microemulsion method. The silica shell growth using the Stöber method did not significantly change the colloidal properties of the dispersion, which is shown by the z-average value and the relatively narrow PDI value of sample C1_5S. The z-average value exceeds the average diameter derived from TEM by (17 ± 5)%. The dispersion has a high colloidal stability due to the highly negative zeta potential of the particles, which is typical for silica dispersions from Stöber-like growth processes containing particles of similar sizes [58,60–61]. Since the surface of the particles corresponds to that of particles from a standard Stöber synthesis, the colloids can be functionalized with the same diverse methods as Stöber silica particles [58].

Figure S5 in Supporting Information File 1 shows X-ray diffraction (XRD) measurements of the oleate-coated UCNP cores C1 and the same particles after the growth of two, three, four and five additional silica shells (samples C1_2S, C1_3S, C1_4S, and C1_5S). These data exclude a possible influence of the silica shell on the crystallinity of the UCNP core. The cores have a predominantly hexagonal crystal structure. Minor peaks at 47° (220) and 55° (311) 2θ indicate a small fraction of the cubic phase. The XRD patterns of the silica-coated UCNPs show the same peaks (mainly the hexagonal phase) with decreasing intensity as the silica shell thickness increases. Accordingly, the broad signal of the amorphous silica at 2θ = 20–25° becomes more dominant with increasing thickness of the silica shell. These data indicate that the crystal structure of the UCNP cores is not changed during the silica shell formation process.

**Figure 3:**
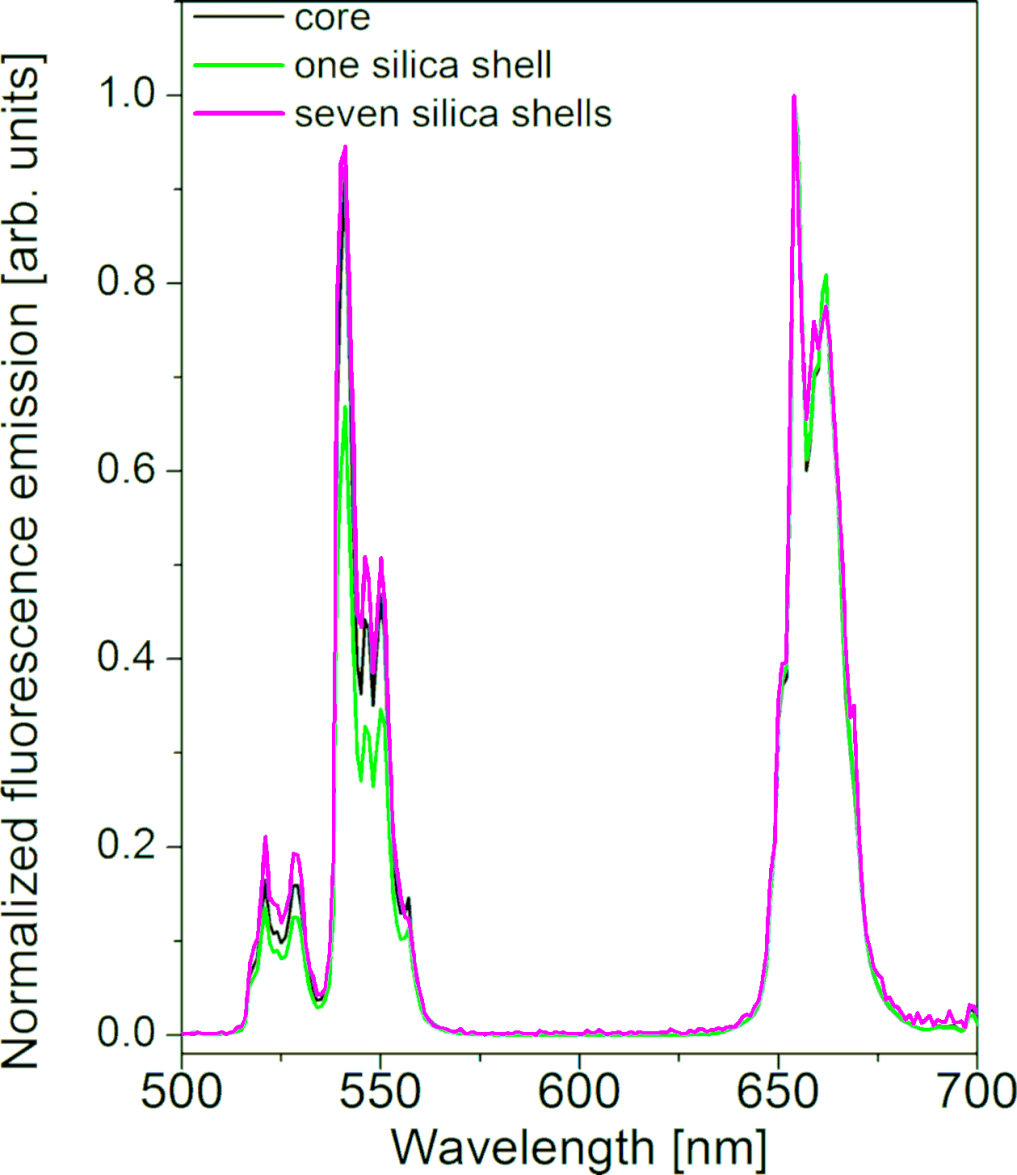
UCL spectra of the oleate coated UCNP cores C2 (20 ± 2 nm diameter, black line) in cyclohexane and after coating with one silica shell (sample C2_1S, shell thickness: 11 ± 1 nm, green line) and seven shells (sample C2_7S, shell thickness: 61 ± 1 nm, pink line) in ethanol. All spectra are normalized at 655 nm for better comparison. The excitation power density was 2 W/cm^2^ at 980 nm.

Figure 3 shows the upconversion luminescence (UCL) spectra of the UCNP cores before the growth of a silica shell (sample C2) and after coating with one (sample C2_1S, shell thickness: 11 ± 1 nm) and seven silica layers (sample C2_7S, shell thickness: 61 ± 1 nm). Sample C2_7S corresponds to the final product with a thick silica layer produced in a modified Stöber growth. All spectra show the typical green and red Er^3+^ emission bands of NaYF_4_:(Yb,Er) UCNPs [62–64]. The silica coating only slightly alters the relative spectral distribution of the UCL spectra. The most pronounced effect is the slight reduction of the green emission bands at 520 and 540 nm of the UCNPs with the thinnest silica shell (sample C2_1S) compared to the oleate-functionalized particles. Similar effects have been reported previously for silanized UCNPs after their transfer into water [65]. They can be explained by the presence of UCL quenching by high-energy vibrators such as -OH groups from ethanol and maybe also from silanol or silanolate groups of the silica network. The increase in non-radiative relaxation processes by surface quenching effects caused for example by Igepal CO-520 after silica coating can lead to a decrease of the UCL intensity [66].

The considerable influence of such quenchers on UCL spectral distribution and UCL quantum yield has been previously shown by us by comparing the excitation power density-dependent UCL of bare UCNPs in organic solvents, water, and D_2_O [67]. A further increase in silica shell thickness barely alters the red-to-green intensity ratio. Here it needs to be kept in mind that the different sizes of the silica-coated particles can affect their scattering characteristics and thereby the excitation power density distribution within the optical cell used for the UCL measurements. In the case of UCL, which depends on the excitation power density, this can influence both the UCL intensity and the UCL spectral distribution. In general, the coating with a thick silica shell is not expected to strongly affect the brightness of the UCNPs as long as the two properties absorption cross section and fluorescence quantum yield, which determine the particle brightness, are not considerably affected. Nevertheless, it must be kept in mind that the increased scattering originating from larger particles can affect the excitation power density that is effective on the dispersed UCNP. As the emission spectra and the relative spectral distribution of UCL both depend on the excitation power density, this can in principle also affect the relative intensity ratios of the green and red emission bands [68–69].

## Conclusion

A concept for the growth of silica shells of sizes between 5 and 250 nm on oleate-stabilized UCNPs was developed. This concept comprises (1) the growth of an initial 5–11 nm thick shell on the oleate-stabilized particles in a reverse microemulsion using the surfactant Igepal CO-520 and an ammonia water concentration of 1.7 ± 0.5 wt % and (2) a further stepwise growth of these particles in the same reverse microemulsion without any intermediate isolation or purification steps of the nanoparticles. In each step, the same volumes of TEOS and ammonia water were added, and the volumes of cyclohexane and Igepal CO-520 were increased so that the ammonia water concentration in cyclohexane was 3.3 ± 0.1 wt %, and that of Igepal CO-520 in cyclohexane was kept constant at 14 ± 1 wt %. In this way, the number of micelles remained constant to match the number of UCNP cores. Also, the micelle size was adjusted to ensure that they were large enough to host the growing core–shell particles. Simultaneously, the aqueous domain was kept small enough to prevent the formation of core-free silica particles. In this stepwise procedure, the zeta potential of the particles becomes increasingly more negative. When the zeta potential of the silica-coated UCNPs reached −40 mV, the particles which then had a silica shell thickness of about 40–50 nm could be dispersed in an ammoniacal ethanol solution with a rather high ammonia water concentration (12–13 wt %) and could be grown by continuous addition of TEOS in one step up to a diameter of more than 500 nm in a modified Stöber process. This stepwise procedure was necessary for growing thick silica shells on the UCNPs since a direct growth of silica on oleate-functionalized UCNPs was not possible in a Stöber-like growth process affording NPs with a hydrophilic surface. A Stöber growth of a silica shell on the UCNPs coated with only a thin silica shell leads mainly to coalesced multicore particles. The latter is related to the relatively small zeta potential of these silica-coated UCNPs which are not very stable in ammoniacal ethanol. Despite the rather harsh conditions during the growth process, this procedure does not influence the crystal structure of the UCNPs and the shape of the UCL emission spectra. This stepwise shell growth can most likely be also utilized for the coating of other NPs with similar hydrophobic surface chemistries of the initial particles such as iron oxide NPs or semiconductor NPs. Further applications can include the covalent attachment of biomolecules such as peptides, antibodies or nucleic acids for bioimaging applications or fluorescence assays. The growth of a mesoporous silica shell on a microporous silica shell can also be applied for the subsequent use of these nanomaterials for drug loading and delivery [69].

## Experimental

All syntheses were carried out with standard glass equipment. The reaction vessels were cleaned before use with hydrofluoric acid (8 vol %) and were then repeatedly rinsed with water. The redispersion of the nanoparticles was carried out using an ultrasonic bath (Sonorex RK512H (860 W, 35 kHz) from Bandelin). Alternatively, a sonotrode UP200H (200 W, 24 kHz) from Hielscher was used. Ultrapure water (Millipore; filter size = 0.22 μm, ρ = 18.2 MΩ cm) was used for all syntheses. For the controlled addition of TEOS, a peristaltic pump (REGLO Digital MS–2/8–160) from Ismatec with a TYGON R-3603 tubing, type AME-01or an LA-30 syringe pump from Landgraf Laborsysteme HLL GmbH was used.

### Materials

Cyclohexane (tech. 99.5%) and ammonia water (p.a., 25 wt % NH_3_) were purchased from Roth. Oleic acid (OA, 90%), erbium chloride hexahydrate (ErCl_3_·6H_2_O, 99.9%), ytterbium chloride hexahydrate (YbCl_3_·6H_2_O, 99.9%) and yttrium chloride hexahydrate (YCl_3_·6H_2_O, 99.9%) were received from ABCR. Sodium hydroxide (NaOH, 99%) was obtained from Grüssing, Ethanol (EtOH, 100%) from Berkel AHK and hydrofluoric acid (HF, 30%) from Riedel de Haën. Polyoxyethylene (5) nonylphenyl ether (Igepal^®^ CO-520), ammonium fluoride (NH_4_F, 99.8%), 1-octadecene (tech. 95%), sodium oleate (82%), tetraethyl orthosilicate (TEOS, 98%) as well as yttrium-, ytterbium- and erbium standards for inductively coupled plasma optical emission spectroscopy (ICP-OES) measurements (TraceCERT^®^, *c* = 1000 mg/mL) were purchased from Sigma Aldrich. All chemicals were used without further purification.

### Synthesis

NaYF_4_:(Yb,Er) UCNPs were prepared from the corresponding lanthanide oleates [14,70] according to a modified procedure from Na and co-workers [16]. For details, see Supporting Information File 1.

### Growth of the silica shell

#### Shell growth in a reverse microemulsion

The synthesis of the silica coating was based on a modified microemulsion method [24,71]. The following describes a typical microemulsion synthesis for the silica coating of UCNPs.

For the first silica shell growth with a calculated thickness of 5 ± 1 nm, a dispersion of UCNPs (diameter = 24 ± 2 nm; *c* = 3 g/L in 11 mL of cyclohexane) was mixed with 0.154 mL of Igepal CO-520. After sonication for 10 min, 1.213 mL of Igepal CO-520 was added, and after brief mixing with sonication, 0.159 mL of ammonia water were added, and the dispersion was sonicated for another 20 min. Finally, 0.159 mL of TEOS were added, and the whole mixture was sonicated for at least 1 h. Generally, a concentration of 16 ± 1 wt % in cyclohexane was used for Igepal CO-520, and the ammonia water concentration was 1.7 ± 0.7 wt % (density of ammonia water was 0.90 ± 0.09 g/mL for a concentration of 25 wt % NH_3_) in cyclohexane for the growth of the first shell. Finally, the dispersion was stirred for 12 h at 1200 rpm at room temperature.

For the further stepwise growth of the silica shells, additional cyclohexane, Igepal CO-520 and ammonia water were added sequentially to the non-purified dispersion to obtain a constant surfactant concentration of 14 ± 1 wt % in cyclohexane and a maximal ammonia water concentration of 3.3 ± 0.1 wt % in cyclohexane. TEOS was added gradually with a rate of 20.8 μL/min through a peristaltic pump while the dispersion was stirred for 12 h at 1200 rpm at room temperature. Alternatively, a vortex shaker from Scientific Industries, Inc. (Model no. G560E) was used.

It was important that the microemulsion was not broken during the entire synthesis, i.e., the last layer of silica was grown before the particles were precipitated with ethanol. In Table S1 in Supporting Information File 1, an example of the amounts of solvent and reactant for a typical multi-step silica shell synthesis with the reverse microemulsion method is given. Table S2 summarizes the surfactant concentration (*c*(Igepal)), ammonia water concentration (*c*(ammonia water)) and the ammonia water-to-Igepal CO-520 mass ratio in each growth step of the silica shell.

After the last growth step, the particles were precipitated by adding 5–10 mL of EtOH and washed three times by repeated centrifugation (1200*g*, 1 h) and redispersion in 10 mL of EtOH and finally redispersed in 10-15 mL of EtOH.

#### Shell growth via a modified Stöber approach

The growth of silica shells on UCNPs via a modified Stöber method [25,28] was carried out after the multistep growth of silica shells (shell thickness = 40–50 nm) with the reverse microemulsion approach. At this point, the zeta potential of the silica particles reached a value below −40 mV in water at pH 7.

In a typical reaction (growth from 112 ± 4 nm diameter to an intended diameter of 300 nm), 2 mL of ammonia water were added to 16.25 mL of an ethanolic dispersion of silica-coated UCNPs (*c* = 1 g/L). Subsequently, 1.1 mL of TEOS were added dropwise with the help of a peristaltic pump (*v* = 20.8 µL/min) to this mixture under magnetic stirring (600 rpm). After the addition was completed, the reaction mixture was stirred for another 12 h. Then, the particles were washed three times by centrifugation (3300*g*, 1 h) and redispersion in 10–20 mL of EtOH with the help of an ultrasonic bath and were finally redispersed in 10 mL of EtOH.

### Characterization

#### Scanning transmission electron microscopy (STEM)

STEM images were recorded with a Hitachi SU 8030 scanning electron microscope in STEM mode with an electron acceleration voltage of 30 kV and a current of 20 µA. A droplet of a dispersion (*c* = 0.5–1 g/L) of the particles in either cyclohexane for oleate-functionalized UCNP cores or ethanol for silica-coated UCNPs was dried on a carbon-coated copper grid (Cu 400 mesh, Quantifoil^®^: 100 carbon support films). The images analysis was carried out with the software FIJI.

#### Dynamic light scattering (DLS) and electrophoretic light scattering

The DLS measurements were carried out with a Zetasizer Nano ZS from Malvern Instruments at 25 °C using a wavelength of 633 nm. The uncoated cores were dispersed in cyclohexane, and the silica-coated particles were dispersed in ethanol and filtered with a sterile syringe filter (pore size: 0.2 µm; materials: nylon or polytetrafluoroethylene (PTFE) for particles dispersed in cyclohexane and nylon or regenerated cellulose for particles dispersed in ethanol, Rotilab). Zeta potential measurements of the aqueous dispersions were carried out with a Zetasizer Nano ZS in capillary zeta cells DTS 1070 from Malvern Instruments. The concentration of the samples in all measurements was between 0.5 and 1 mg/mL.

#### Measurements of the upconversion luminescence (UCL)

The UCL measurements were carried out with a FluoroMax-4 spectrometer from Horiba Jobin Yvon equipped with a 2 W 980 nm laser diode from Insaneware-Robert Nowak and an Edinburgh Instruments spectrofluorometer FLS-980 equipped with an electrically modulated 8 W 978 nm laser diode (950 μs long square pulses) and a red-extended photomultiplier tube (Hamamatsu R2658P). Quartz glass cuvettes (QS Suprasil, 5 mm, Hellma or VWR) were used in all measurements performed at room temperature. The concentration of the samples was 1–2 g/L in cyclohexane for oleate-capped UCNPs or ethanol for silica-coated UCNP.

#### Inductively coupled plasma-optical emission spectroscopy (ICP-OES)

For the determination of the elemental composition of the UCNP samples, 1 mL of the dispersions (*c* = 5 g/L in cyclohexane for the oleate-coated UCNPs or in ethanol for the silica-coated UCNP) was dried. The dried particles were dissolved in 1 mL of aqua regia for at least 30 min and diluted with at least 5 mL of ultrapure water. The measurements were carried out using an iCAP 6000 Series ICP Spectrometer from Thermo Scientific with a radial optical approach. A series of solutions with different concentrations were prepared separately for calibration from an yttrium standard for ICP (*c*(Y^3+^) = 10, 20 and 40 ppm), ytterbium standard for ICP (*c*(Yb^3+^) = 10, 20 and 40 ppm) or erbium standard for ICP (*c*(Er^3+^) = 1, 5 and 10 ppm).

#### X-ray diffraction (XRD) measurements

A minimum amount of 10 mg of dried particles were used for the XRD measurements. The XRD device was a Rigaku SmartLab 3 kW with a DTex Ultra 250 detector (40 kV, 30 mA) equipped with a Cu Kα_1_ radiation source and a radiation wavelength of 0.15405 nm. The angle range of the measurements was 10–60° 2θ, measurement time was 60 s/0.3°.

## Supporting Information

File 1 Synthesis details, additional STEM images, and XRD data.
