# Supplementary material for: Coating of upconversion nanoparticles with silica nanoshells of 5–250 nm thickness
Source: Beilstein J Nanotechnol. 2019 Dec 9;10:2410–21. doi: 10.3762/bjnano.10.231 (PMC6941407; doi:10.3762/bjnano.10.231)
Supplement: File 1 — Synthesis details, additional STEM images, and XRD data. [file Beilstein_J_Nanotechnol-10-2410-s001.pdf]

BEILSTEIN JOURNAL OF NANOTECHNOLOGY

# Supporting Information

for

# Coating of upconversion nanoparticles with silica nanoshells of 5–250 nm thickness

Cynthia Kembuan, Maysoon Saleh, Bastian Rühle, Ute Resch-Genger and Christina Graf

*Beilstein J. Nanotechnol.* **2019,** *10,* 2410–2421. doi:10.3762/bjnano.10.231

# Synthesis details, additional STEM images, and XRD data

License and Terms: This is a supporting information file under the terms of the Creative Commons Attribution License (http://creativecommons.org/licenses/by/4.0). Please note that the reuse, redistribution and reproduction in particular requires that the authors and source are credited.
The license is subject to the *Beilstein Journal of Nanotechnology* terms and conditions: (https://www.beilstein-journals.org/bjnano)

## Synthesis of lanthanide oleates as precursors for the synthesis of upconversion nanoparticles

The synthesis procedure was modified from a literature approach for the preparation of iron oleate as a precursor for the synthesis of iron oxide nanoparticles [1,2]. As an example, the synthesis of $NaYF_4$ nanoparticles doped with 18% Yb and 2% Er from 1 mmol of lanthanoide chlorides is described: A mixture of yttrium chloride hexahydrate (0.242 g; 0.78 mmol), ytterbium chloride hexahydrate (0,069 g; 0.18 mmol), and erbium chloride hexahydrate (0.006 g; 0.02 mmol) was dissolved in 3 mL of ultrapure water, 7 mL of ethanol (EtOH) and 7 mL of hexane. Sodium oleate (1.22 g; 4 mmol) was dissolved in a mixture of 2 mL of ultrapure water and 3 mL of ethanol in a 50 mL one-necked flask, then the solution of the mixed lanthanide chlorides was added under stirring. Another 7 mL of hexane were finally added under stirring, and the solution was heated to reflux at 70 °C for 4 h. The solution was then cooled to room temperature, and the organic phase was extracted three times with 20 mL of ultrapure water. Subsequently, the organic phase was separated, volatiles and hexane were removed in vacuo, and the waxy white product was dried under vacuum ($p = 5 \times 10^{-3}$ mbar) in a 100 mL three-necked flask at room temperature before being used in the next step without further purification.

## Synthesis of upconversion nanoparticles

The following method is the typical synthesis procedure for 1 mmol $NaYF_4$:(Yb,Er) nanoparticles from rare earth oleates [3]. The rare earth oleates from the above described method were dissolved in a mixture of 15 mL 1-octadecene and 7 mL oleic acid in a three-necked flask with a heating mantle with an automatic temperature controller under an inert atmosphere of argon, heated under vacuum until the reaction mixture reached 100 °C and kept at 100 °C for 1 h to evaporate all water and hexane. The mixture was cooled down to 50 °C under argon flow. NaOH (100 mg; 2.5 mmol) and $NH_4F$ (148 mg; 4 mmol) were dissolved in 5 mL of MeOH and added to the oleates with a syringe. The mixture was stirred for 30 min at 50 °C at 300 rpm. Afterward, MeOH was evaporated under reduced pressure ($p = 5 \pm 1 \times 10^{-3}$ mbar). After three vacuum degassing and argon flushing cycles to ensure that all MeOH has been evaporated, the reaction mixture was heated to 300 °C and stirred at 500 rpm at this temperature under argon for 1.5 h. After the reaction mixture was cooled down, 15–20 mL of EtOH were added to precipitate the particles. The white powder was redispersed in cyclohexane, precipitated with 5–10 mL of EtOH and centrifuged at 1100*g* for 15 min. This redispersion/reprecipitation process was repeated three times. The particles were finally redispersed and stored in 10 mL of cyclohexane.

**Table S1:** Amount of reactants for a stepwise silica coating of UCNP cores with the microemulsion method.

| Layer | Thickness of the existing $SiO_2$ shell (calculated) [nm] | Calculated growth of the $SiO_2$ shell [nm] | Total thickness of the $SiO_2$ shell [nm] | Volume of initial dispersion [mL] | Added volumes | | | |
|---|---|---|---|---|---|---|---|---|
| | | | | | Cyclohexane [mL] | Igepal [mL] | Ammonia water [mL] | TEOS [mL] |
| 1 | 0 | 7 | 7 | 0.781 | 11.00 | 1.364 | 0.159 | 0.159 |
| 2 | 7 | 11 | 18 | 13.463 | 16.85 | 1.850 | 0.674 | 0.674 |
| 3 | 18 | 14 | 32 | 33.511 | 66.05 | 7.29 | 1.887 | 1.887 |
| 4 | 32 | 11 | 43 | 97.162 | 93.10 | 10.272 | 2.660 | 2.660 |

**Table S2:** Amount of surfactant, ammonia water concentration, and ammonia water-to-Igepal weight ratio used in the microemulsion synthesis for each sample.

| **Sample** | **Shell** | ***c* (Igepal) [wt % in cyclohexane]** | ***c* (ammonia water) [wt % in cyclohexane]** | **Ammonia water-to Igepal weight ratio** |
|---|---|---|---|---|
| C1_1S | 1st | 15.90 | 1.67 | 1:9.5 |
| C1_2S | 2nd | 14.79 | 3.45 | 1:4.3 |
| C1_3S | 3rd | 14.32 | 3.34 | 1:4.3 |
| C1_4S | 4th | 14.22 | 3.31 | 1:4.3 |

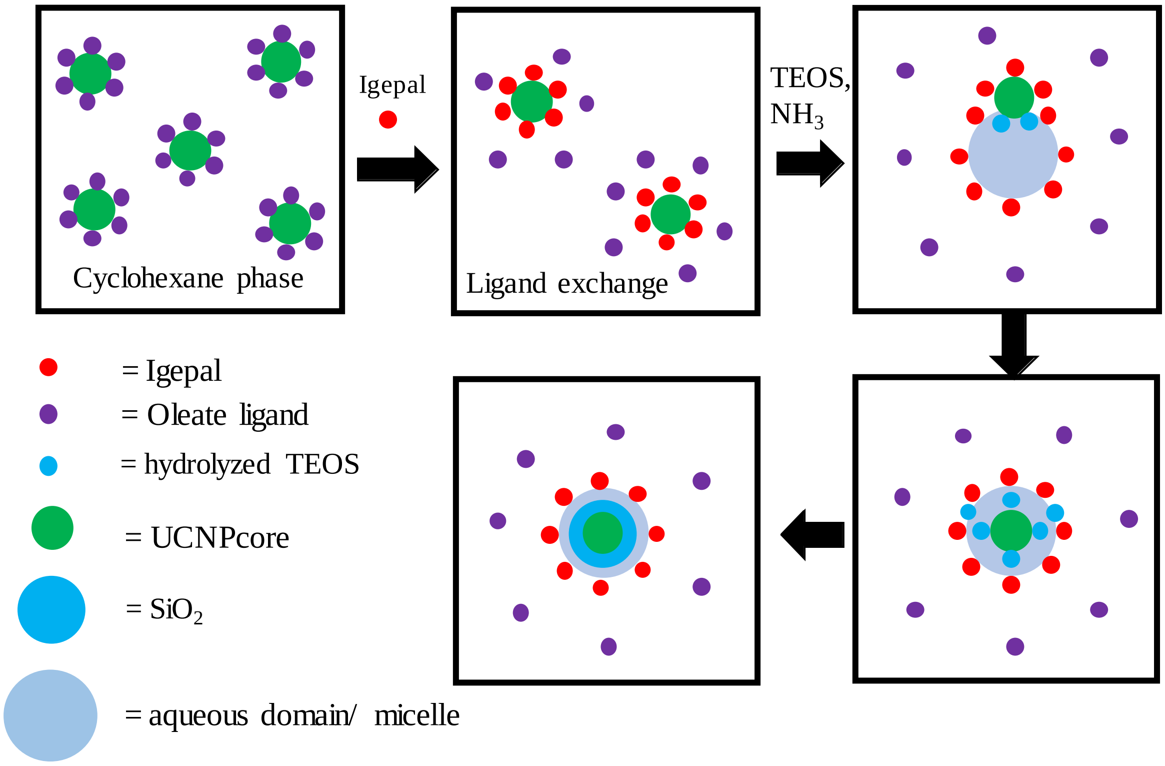


**Figure S1:** Scheme of microemulsion synthesis for coating oleate-functionalized UCNP cores with a first thin silica shell. Upon addition of surfactant to the UCNP dispersion, a ligand exchange between oleate ligands and the surfactant occurs. Upon addition of TEOS and $NH_3$, aqueous micelles are formed, and TEOS is hydrolyzed at the interface between the oil (cyclohexane) and the water phase. A second ligand exchange occurs between hydrolyzed TEOS (silica monomers) and Igepal, which transfers the UCNP into the micelles where the condensation reaction of the silica monomers take place, leading to the formation of the final silica shell.

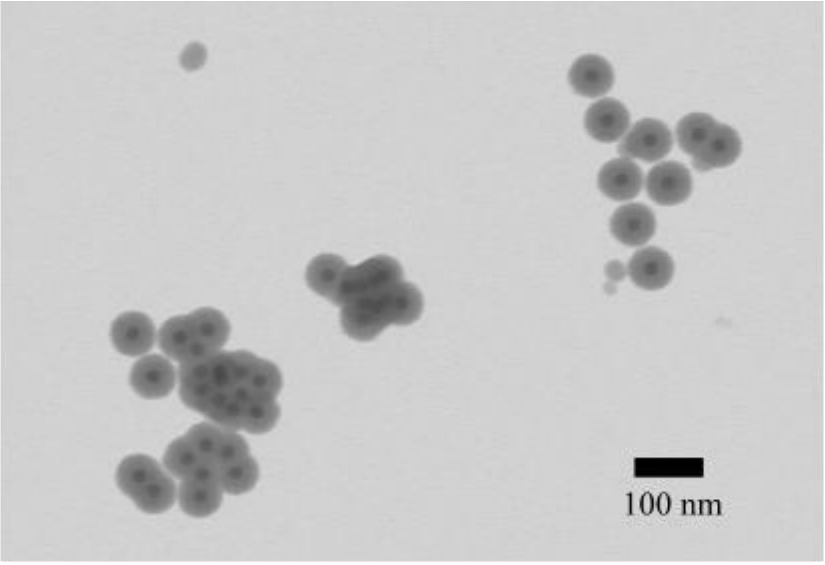


**Figure S2:** STEM image of UCNPs (sample C2, 20 ± 2 nm diameter) that were coated in a Stöber-like growth process after an initial thin (11 ± 1 nm) silica shell was grown in a reverse microemulsion.

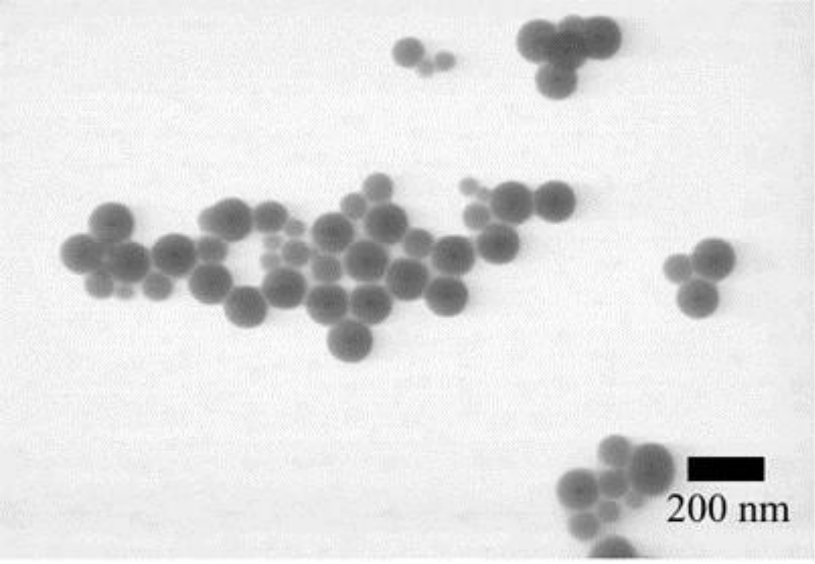


**Figure S3:** STEM image of UCNPs (sample C1, 24 ± 2 nm diameter) that were coated in a four-step silica shell process in a reverse microemulsion process with an R-value of 1:2.2, which leads to the formation of core-free silica particles due to a too high ammonia water concentration, which accelerates the hydrolysis rate of TEOS. The size of the silica-coated UCNPs was 107 ± 6 nm, and the core-free particles had an average diameter of 60 ± 15 nm.

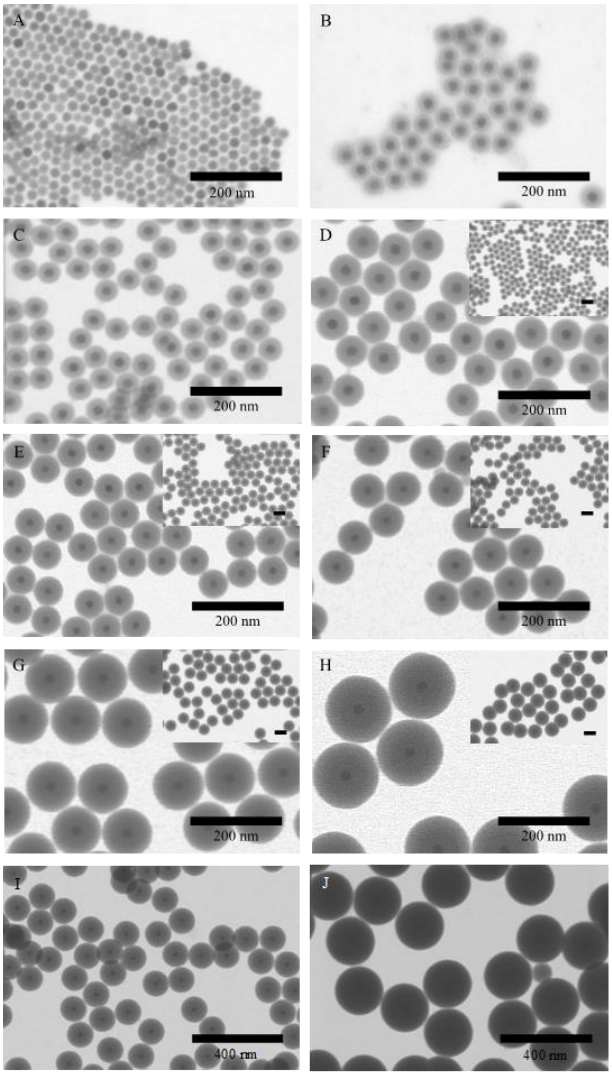


**Figure S4:** STEM images of oleate-stabilized (A) UCNP cores (sample C2, 20 ± 2 nm diameter) coated by an optimized reverse microemulsion method with (B) one silica shell (sample C2_1S, 11 ± 1 nm thickness), (C) two silica shells (sample C2_2S, 16 ± 1 nm thickness), (D) three silica shells (sample C2_3S, 24 ± 1 nm thickness), (E) four silica shells (sample C2_4S, 31 ± 1 nm thickness), (F) five silica shells (sample C2_5S, 38 ± 1 nm thickness) and (G) six silica shells (sample C2_6S, 43 ± 1 nm thickness). The final seventh layer in the Figures (H), (I) and (J) was grown by a modified Stöber process (sample C2_7Sa, 61 ± 1 nm shell thickness, sample C2_7Sb, 48 ± 3 nm shell thickness, and C2_7Sc, 95 ± 4 nm shell thickness). The scale bar in the insets of panels (D–H) represents 200 nm, while the scale bar in the insets of Figure (I) and (J) represents 400 nm.

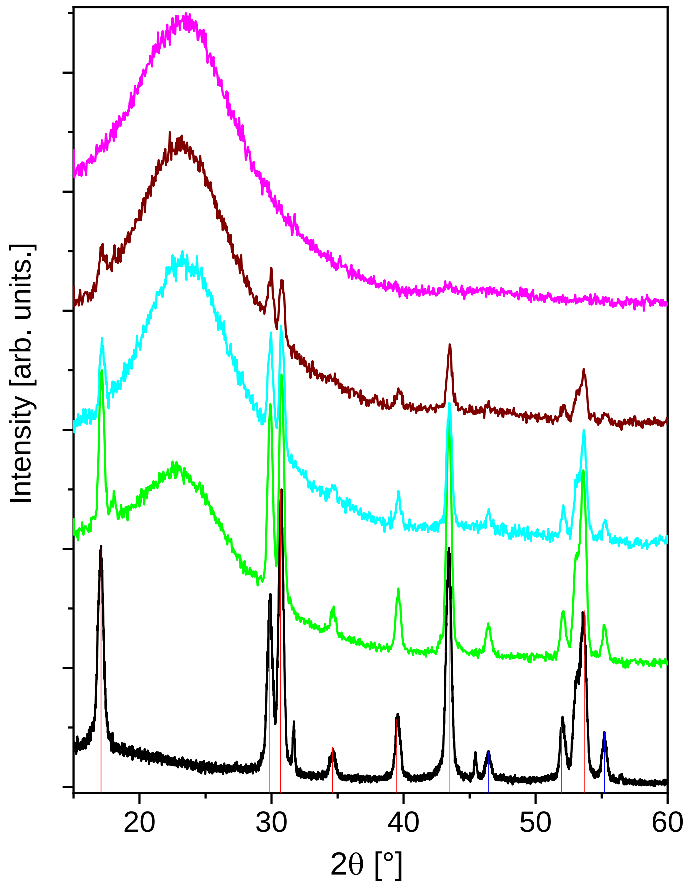


**Figure S5:** XRD patterns of oleate-coated UNCP cores (24 ± 2 nm diameter, sample C1, black line) where red bars indicate reference values of the hexagonal phase (JCPDS No. 00-028-1192) and blue bars indicate reference values of the cubic phase (JCPDS No. 01-077-2042) and XRD patterns of the same cores after the second silica coating (C1_2S, shell thickness: 18 ± 2 nm, green line), third silica coating (C1_3S, shell thickness 35 ± 2 nm, cyan line), fourth shell silica coating (C1_4S, shell thickness: 44 ± 2 nm, brown line) and fifth silica coating (C1_5S, shell thickness: 149 ± 8 nm, pink line).

## References


1. Park, J.; An, K. J.; Hwang, Y. S.; Park, J. G.; Noh, H. J.; Kim, J. Y.; Park, J. H.; Hwang, N. M.; Hyeon, T. *Nat. Mater.* **2004**, *3*, 891–895,
2. Wei, Y.; Lu, F. Q.; Zhang, X. R.; Chen, D. P. *Chem. Mater.* **2006**, *18*, 5733–5737.
3. Na, H.; Woo, K.; Lim, K.; Jang, H. S. *Nanoscale* **2013**, *5*, 4242–4251.
